# From passive consumers to critical evaluators: promoting media literacy in animal science

**DOI:** 10.1093/tas/txag073

**Published:** 2026-06-05

**Authors:** Ciana M Bowhay

**Affiliations:** School of Agriculture, Tennessee Technological University, 715 Quadrangle Cookeville, TN 38505, United States

**Keywords:** media literacy, misinformation, animal science education, transformative learning, digital communication

## Abstract

Media literacy is increasingly vital in animal science education, where students face widespread misinformation about production practices, nutrition, and animal health and welfare. Students often overestimate their ability to discern credible sources from misinformation which can create barriers to learning when misconceptions interfere with integration of new knowledge. This study examined how an assignment grounded in principles of the critical media literacy framework influenced students’ evaluation of credibility and perceptions of misinformation. This assignment was implemented in two semesters of an undergraduate Animal Nutrition course (*n* = 42) and required students to critically evaluate media related to course content. Student assignments and reflections were analyzed using qualitative open coding to identify emerging themes. Students indicated that prior to engaging in the assignment, they relied on superficial indicators of credibility like perceived author expertise, presentation style, or popularity. After completing the project, students reported greater awareness of bias and misinformation and commitment to deeper evaluation strategies when consuming media. Many indicated intentions to research claims more thoroughly, approach popular media about animal science with increased skepticism, and assess the credibility of authors before accepting their claims. These findings suggest that integration of media literacy activities in animal science curricula can support critical reflection practices on students’ evaluation of information. This provides conditions consistent with transformative learning by prompting students to challenge existing assumptions and adopt a more reflective approach when navigating digital media. Developing these skills is essential for future animal science professionals to critically evaluate information and engage credibly in scientific discourse within a rapidly evolving digital landscape.

## Teaser

This study demonstrates how integrating critical media literacy principles into animal science education helps to equip students to evaluate misinformation and think critically about digital content. These skills are essential for future animal science professionals who will need to communicate credible, evidence-based information in an increasingly digital world.

## Introduction

### Prevalence of misinformation

Since 2000, there has been a dramatic shift from traditional to digital media consumption (Guttmann 2023). The average undergraduate in America now spends a significant portion of their day engaging with digital content, with much of that time spent on social media (Clinton 2025; Harmony Healthcare IT 2025). This shift towards digital media sources has resulted in a sharp increase in available information and perspectives, where anyone with an internet connection can create and share content (Jungherr et al. 2020).

Learning to evaluate information sources is a valuable life skill for all students, particularly in agriculture, where misinformation runs rampant (Woodside 2024). In line with global information trends, consumers are increasingly reliant on digital sources like social media or online articles and blogs for agricultural and nutrition information (Verbeke 2005; Capper and Yancey 2015; Fischer et al. 2020). Unfortunately, misinformation spreads more rapidly than truth (Vosoughi et al. 2018; Pierri and Ceri 2019). Poor media literacy can contribute to misconceptions and poor decision making based upon erroneous information.

Pre-existing misconceptions present a barrier to meaningful science learning as students rely on existing knowledge to comprehend and assimilate new ideas (Eaton et al. 1984; Köse 2008; Kellner and Share 2019). If that knowledge is false or biased, new information becomes difficult to integrate, especially if it contradicts existing beliefs (Lewandowsky et al. 2012).

Despite warranted concern about misinformation in agriculture, few studies have examined how structured media literacy interventions influence students’ evaluation of digital information in animal science. For animal science graduates, the ability to critically evaluate and communicate scientific information extends beyond academic success. Many animal science students will enter public-facing career paths like extension, veterinary practice, or industry roles that require engagement with producers, consumers, and the public. Animal science professionals must navigate digital environments and address misinformation related to animal production, nutrition, health, and welfare (Capper and Yancey 2015). Developing media literacy skills as an undergraduate is therefore essential for preparing students to function effectively as credible communicators (Brownell et al. 2013).

### Critical media literacy framework

Media literacy is an easily overlooked aspect of education that has become imperative in today’s information landscape. Critical media literacy (CML) extends beyond understanding and interpreting media; it requires evaluators to consider the wide variety of factors that influence both its creation and interpretation (Halpern 2024). With this in mind, Kellner and Share (2019) introduced a critical media literacy framework to guide learners in addressing these varied factors. This framework asks learners to consider media as fundamentally biased and intentionally designed to achieve a given purpose (Funk et al. 2016; Kellner and Share 2019). Media presents a message shaped by the choices, perspectives, and intentions of the creators. It is also subject to the interpretation of the audience who carry their own prior knowledge, experiences, or biases. The CML framework challenges students to analyze choices, perspectives, and purposes of creators, and consider whose voice is unrepresented, or what context is absent (Kellner and Share 2019). These self-reflective practices lend themselves to transformative learning.

### Transformative learning

Transformative learning takes place when learners encounter views that challenge their perspectives of self or their preconceptions and undergo a shift in perspective, leading to shifts in future actions (Mezirow 2000, 2018; Cranton 2010). According to transformative learning theory, learners may encounter situations that challenge underlying assumptions that shape their ways of constructing meaning. When these assumptions are challenged, learners may engage in critical reflection to reconstruct the way they develop meaning (Mezirow 2018). As it pertains to the CML framework, learners must recognize their existing preconceptions and critically evaluate those of the media creator. Through this process students transform from passive consumers of information to reflexive and purposeful evaluators, less likely to be influenced by misinformation (Funk et al. 2016; Mezirow 2018).

While there is prior literature addressing misinformation in digital communication and media literacy across multiple disciplines, existing work in agriculture and animal science is centered on public perceptions of animal production and prevalence of misinformation in food and agricultural systems. Media literacy has not yet been explicitly studied in animal science. The objective of this study was to examine how a media literacy assignment grounded in CML principles influenced critical thinking, awareness of misinformation, and perceptions of students’ information evaluation practices.

## Methods

All procedures and data collection were approved by the Tennessee Tech University Institutional Review Board, 2670.

### Media literacy project design and data collection

This project was designed to promote media literacy and critical thinking in animal science students and to answer the question “How do students describe their evaluation of media credibility and perceptions of misinformation after engaging in an assignment grounded in the CML framework?” For the purposes of this assignment, *media* was defined broadly as publicly accessible digital content related to course-relevant content including social media posts, short-form videos, blogs, news articles, or other popular press sources.

The media literacy assignment itself was evaluated, in addition to a retrospective reflection, to assess the impact of this learning activity. This study used a qualitative exploratory design to examine student reflections and artifacts generated from a course-based media literacy assignment. The purpose of this study was to document patterns in how students articulated changes in their evaluation of digital media following structured engagement with CML principles.

This study was conducted across two fall semesters in two sections of an undergraduate Animal Nutrition course at a small, primarily undergraduate institution. The media literacy assignment was implemented using the same prompts and instructional framework in both course sections. Total participation across both semesters was *n* = 42 students, reflecting typical class sizes at the institution.

The assignment was administered once per semester and served as a structured learning activity within the course rather than as a repeated intervention. Students were assigned a project grounded in principles of the Critical Media Literacy Framework developed by Kellner and Share (2019). Students submitted their topic and media source mid-semester, with a final draft due at the end of the semester. Reflections were completed following submission of the final draft, at which time students were informed of the study and provided informed consent for the use of their reflection responses and papers. Participation in the research component was voluntary and had no impact on course grading.

This assignment asked students to identify a piece of easily accessible media (popular press, social media, etc.) and evaluate it using guided questions based on the CML framework. Assignment prompts guided students to examine media purpose, intended audience, evidentiary support, framing, and underlying assumptions, while also reflecting on how their own prior background and prior beliefs shaped their interpretation of information. In this way, the assignment served as an instructional scaffold grounded in principles of the CML framework (Kellner and Share 2019). Rather than asking students simply to judge whether media was “correct” or “incorrect,” the prompts were structured to model CML practices by emphasizing source credibility, evaluation of evidence, and the context in which the media was produced and consumed.

Prompts within the assignment were as follows: (1) How have the people around you and the media you consume affected the way you think about nutrition, animal production, or your personal practices? (2) What kind of information about livestock production or nutrition is most readily available? (3) Where did the claims in your piece of media come from, what information (if any) are they based on? (4) Are the claims true? (5) How would you address these claims using evidence from the course, textbook, or other credible resources (peer-reviewed articles or government databases like the EPA). (6) How has this assignment impacted your perspective on the above subject?

To evaluate the effect of a project grounded in media literacy principles on student perceptions and critical evaluation practices, the course instructor also collected student reflections regarding their experiences and perspectives after completing the assignment. Students were asked to reflect and respond to three reflection questions: (R.1) How did you find and select your piece of media? (R.2) Did this project change the way you evaluate media? (R.3) What have you looked for in the past to determine if media is credible or not? Has this changed? Why, or why not? Reflection responses and student papers were then analyzed to identify emergent themes.

### Demographic data

Students who participated in this media literacy project were undergraduates, with a majority being agriculture majors (95.2%) and two biology majors (4.8%). Approximately 93% of students were female. Students taking this course were all in at least the second year of their degree program.

### Data analysis

Student responses were analyzed using the (Chi 1997) 7-step methodology for qualitative analysis. Chi’s (1997) methodology provides a structured approach for analyzing written responses and identifying emergent themes in qualitative educational data, emphasizing iterative coding and careful interpretation of findings in relation to research questions and existing theory. Reflection responses and student assignments were coded using an open coding methodology to identify emerging themes using MAXQDA Analytics Pro (VERBI 2022), and the frequencies of responses evaluated. For the purpose of analysis, descriptors such as *misleading*, *biased*, *extreme*, *agenda-driven*, and *educational* reflect student-generated interpretations rather than researcher-defined classifications. These terms were coded inductively based on language used by students in their written assignments and reflections. As characterized by students, *misleading* or *biased* content was described as information perceived to exaggerate claims, omit context, or promote a particular agenda. Students determined credibility of sources based on responses to questions 3–5 in the assignment prompts listed above. Interpretations reported in the Results represent how students described and made sense of media they encountered.

Open coding resulted in a total of 57 codes and 314 coded segments. Responses to each prompt were tallied and reported. Total student percentages for each response were determined by dividing the frequency of response by the total number of students (*n* = 42). Item response totals differ from the total number of students, as student responses may have fallen under multiple categories, or they may not have provided a response to the prompt in question. Responses that fell under multiple categories were coded separately within each category. Quotes were selected from the top response categories within related prompts to qualitatively illustrate empirical findings.

## Results and discussion

### Media sources and readily available information

Most students (64.3%) selected social media as their information source, with the majority using TikTok. Other media sources cited were YouTube (16.7%), Google searches (11.9%), and news stories or blog posts (7.1%, Table 1).

**Table 1:** Student responses to reflection prompts after completing the media literacy project.

Prompt and response	Frequency Total	Student Total, %^1^
**Where did you find your media piece?**		
**Social Media**	27	64.3
**YouTube**	7	16.7
**Google Search**	5	11.9
**News or Blog**	3	7.1
***Item response total***^2^	*42*	
**How have you evaluated credibility in the past?**		
**Perceived creator credibility**	12	28.6
**Site appears credible**	11	26.2
**Presence of charts, graphs, data**	8	19.0
**Do own research on other sources**	7	16.7
**Confidence of creator, claimed expertise**	6	14.3
**Popularity of post, creator**	5	11.9
***Item response total***	*51*	
**Change in how you evaluate media?**		
**Recognize need for more critical thinking**	12	28.6
**Do own research on topic, creator**	9	21.4
**Realize how easily misinformation spreads**	9	21.4
**No change, already skeptical**	6	14.3
**Yes, no explanation**	5	11.9
**Look for peer reviewed sources**	2	4.8
**Will not be led by emotion**	2	4.8
***Item response total***	*49*	

*Note.* Individual responses to prompts were clustered into categories by theme, counts were made. Responses with frequencies of less than two students were not included in the table.

1 Student percent totals were calculated by taking the response statement count and dividing by total number of students (*n* = 42).

2 Item response totals do not match number of students (*n* = 42) as student responses may fall into more than one category, or they did not respond to the prompt.

When asked what type of information was most readily available, students reported that misinformation, or misleading media about animal production or nutrition (47.6%; Table 2) were the most readily available to them on their chosen platforms. As one student noted,

Misinformation is circling on so many different platforms, it is hard to decipher facts from fiction.

**Table 2:** Frequencies of student’s individual responses and emergent themes by prompt and response in media literacy paper.

Prompt and response	Frequency Total	Student Total, %^1^
**What type of information is most readily available?**		
**Misleading, misinformation**	20	47.6
**Negative press**	9	21.4
**Extreme views, clear agenda or bias**	6	14.3
**Educational resources**	3	7.1
***Item response total***^2^	*36*	
**What was the purpose of the media you evaluated?**		
**Push agenda, sell something**	18	42.9
**Create fear**	9	21.4
**Education**	7	16.6
***Item response total***	*36*	
**How has your perspective changed?**		
**Recognize need for more informed creators**	13	31.0
**Realize proliferation of misinformation**	9	21.4
**Need for critical evaluation, additional research**	9	21.4
**Good learning resources exist outside classroom**	5	11.9
**No change**	2	4.8
***Item response total***	*39*	

*Note.* Individual responses to prompts were clustered into categories by theme, counts were made. Responses with frequencies of less than two students were not included in the table.

1 Student percent totals were calculated by taking the response statement count and dividing by total number of students (*n* = 42).

2 Item response totals do not match number of students (*n* = 42) as student responses may fall into more than one category, or they did not respond to the prompt.

In addition to misinformation, students indicated a predominance of negative press regarding animal production (19.0%), and prevalence of extreme viewpoints or sources with clear agendas or biases (11.9%). Only 7.1% of students reported that they found credible educational resources to be most readily available in their chosen media sources.

As this assignment asked students to find an easily accessible media source to evaluate, it is unsurprising that the majority turned to social media. Throughout the last decade, social media has become an increasingly popular and pervasive information source, largely due to convenience and accessibility (Kim and Sin 2015; Chyne et al. 2023). However, the volume and pace of information availability in digital media environments can make it difficult for users to discern accurate, evidence-based information from misleading or inaccurate claims, particularly when content is presented authoritatively or appeals strongly to emotion (Lewandowsky et al. 2017). While the proliferation of voices and ideas available in the digital age has increased access to diverse ideas, it has also enabled users to curate the content they encounter. Research has shown that personalized content curation in digital media can reinforce existing beliefs and limit exposure to contradictory perspectives (Pariser 2011; Cinelli et al. 2021). Although this study did not examine algorithmic recommendation systems directly, curation of digital media environments provides important context for understanding students’ experiences with media. In their reflections, students described difficulty sorting through misleading information despite availability of credible educational sources.

These observations align with prior research on public and student perspectives of animal agriculture, which suggests that repeated exposure to emotionally charged or misleading content can influence how information is interpreted and make it more difficult to engage with accurate or conflicting evidence when it is presented (Fraser 2001; Bowhay et al. 2022; Kreft et al. 2023). Student reflections also demonstrate awareness of how selective exposure can reinforce existing beliefs, as one student captured succinctly,

When searching for extremely specific and biased information to fit into your idea of right, you will find that information.

These findings underscore the importance of integrating media literacy into animal science curricula to support students in evaluating digital media related to course-relevant topics.

### Purpose of media and credibility

When determining the purpose of their chosen media (Table 2), students most often cited pushing an agenda or selling something (42.9%), or creating fear (21.4%) as the primary goal. Fewer students (16.6%) cited provision of education as the primary aim of their chosen media.

Often media that is commercially motivated or aligned with a particular agenda can be presented in ways that resembles educational content. Producers of this content define a problem or concern and then present solutions linked to specific products, perceptions or actions (Wojdynski and Evans 2016; Woodside 2024). This is evident in public discourse surrounding agricultural practices, nutrition, and animal welfare (Čechmánek 2024). Prior research shows that these strategies often emphasize risk, uncertainty, or critique of conventional practices to advance an agenda or enhance marketing (Frewer et al. 2013). This pattern may be reinforced by digital platforms that favor polarizing content over neutral educational content, as creating a sense of urgency, risk, or promoting a biased narrative drives engagement, which can complicate communication of scientific information (Frewer et al. 2013; Bakshy et al. 2015; Vosoughi et al. 2018; Cinelli et al. 2021). Students’ reflections indicate emerging awareness of agenda-setting processes, as they articulated recognition of marketing claims or bias after completing the guided media analysis. Their ability to recognize these agendas and marketing ploys speaks to engagement in CML principles leading to transformative learning where learners examine existing assumptions about credibility and purpose (Funk et al. 2016; Mezirow 2018; Kellner and Share 2019). The fact that relatively few students assigned an educational purpose free of bias to their media underscores the challenge faced by science communicators who must compete with emotionally compelling narratives to disseminate scientifically accurate information to the public (Brossard 2013). Developing students’ abilities to identify bias and agenda-setting behaviors through this framework is important for future animal scientists who will need to translate scientific knowledge to an increasingly agriculturally illiterate public.

When asked how they judged credibility of sources (Table 1), students indicated that in the past they have relied on surface-level analysis like perceived author expertise (28.6%), site appearance (21.4%), inclusion of sources or graphs and data (19.0%), confidence of the creator (14.3%) or popularity of a post (11.9%). Several students (16.7%) reported that they regularly did their own research of media claims using additional trusted sources like the American Veterinary Medical Association website, or peer-reviewed articles. Student comments illustrate these strategies, stating,

I have found myself believing information if the source is confident in their reasoning.

Another student stated that

…with a large number of likes and views on [a] video, I would assume it was correct information.

These findings concur with previous research on the subject which found that students often rely on simplistic strategies when evaluating online resources (Kim et al. 2021; Zlatkin-Troitschanskaia et al. 2021; Kunz et al. 2024). Although undergraduate students generally rate their ability to evaluate credibility of online sources well, they tend to have poor perception of their personal limitations in identifying unreliable media (Butler Horton 2021). This is likely due in part to the illusory truth effect (Vasu et al. 2018), where the same inaccurate information repeated from multiple sources creates the illusion of credibility (Pennycook et al. 2018). This phenomenon is reported in media literacy literature (Van Bavel et al. 2021), and becomes particularly relevant as students encounter personalized content curated specifically for their interests and opinions (Kümpel 2022; Chyne et al. 2023). Especially when it comes to information gleaned from social media, a more nuanced evaluation strategy is needed (Lewandowsky et al. 2012; Cho et al. 2024).

### Personal takeaways and transformative learning

After completing this project, students reflected on how their manner of evaluating media changed (Table 1). In their reflections they recognized a need for more critical thinking (28.6%) and personal research on the topic and creator (21.4%) when consuming media. Students also reported increased awareness of how easily misinformation spreads (21.4%), with one student stating

Through this project, I was able to discover firsthand just how easy it is for misinformation to spread through media and not be second-guessed by viewers.

Some students explicitly noted intentions to “not be led by emotion” (4.8%) and to seek peer reviewed sources (4.8%). In the words of one student describing how the project shifted their perspective,

If an author is trying to make a valid claim, they need to not only support their argument but also give evidence as to why they are making their claim. This project opened my eyes to be aware of conscious and unconscious bias that is in media that is readily available to everyone.

A subset of students indicated that they will not change how they evaluate media as they already approached it with skepticism (14.3%).

When asked about their personal takeaways or change in perspective after completing this project (Table 2), students recognized a need for more informed creators and evidence-based content (31.0%), stating,

We need more true nutritional scientists speaking up on social media and taking on the forefront of educating the masses.

**Figure 1:**
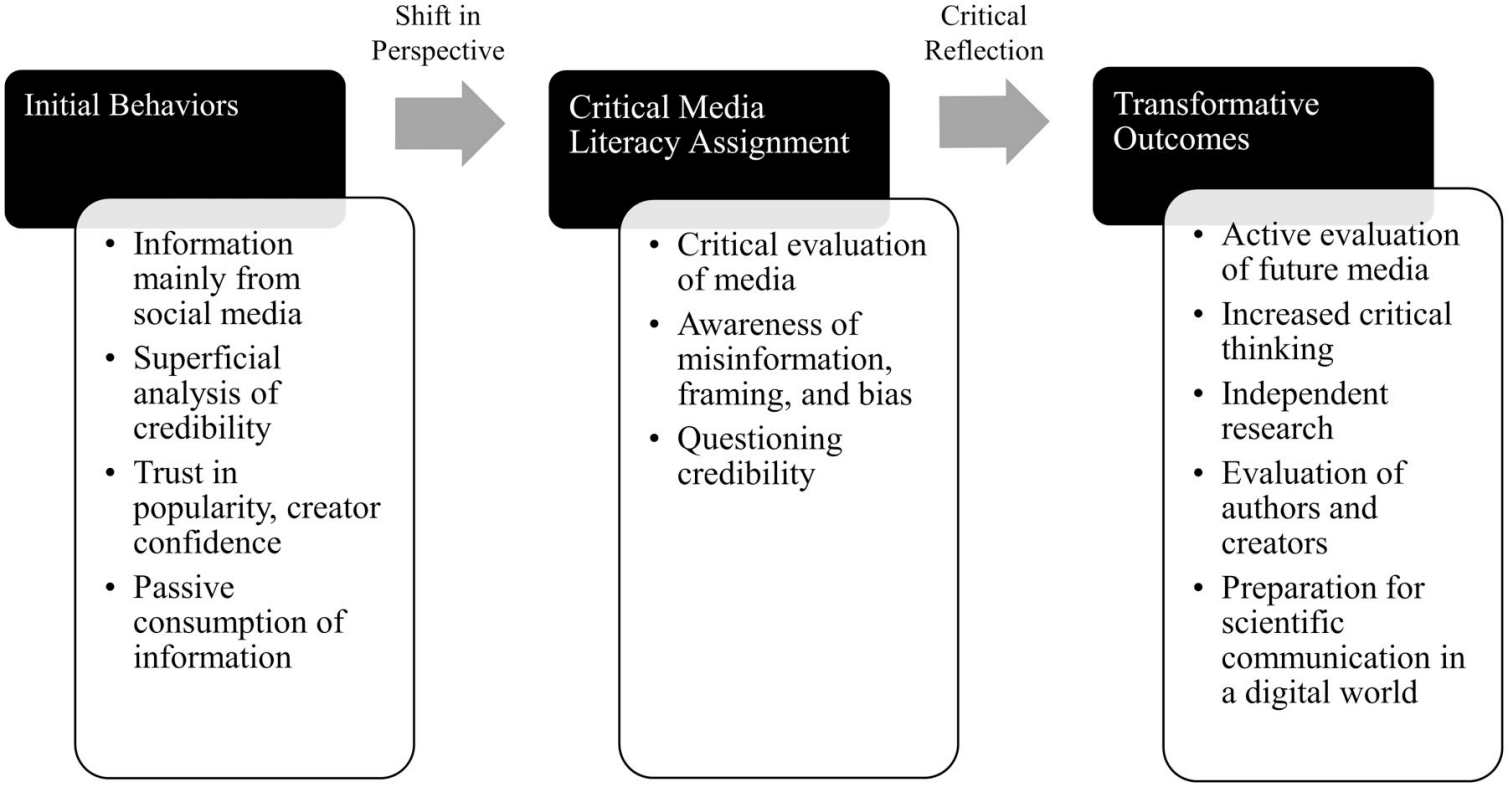
Author-developed conceptual flow diagram illustrating themes identified in student reflections and the reported progression in media evaluation behaviors following completion of a critical media literacy assignment in an Animal Nutrition course.

Several students (11.9%) reported that they found easily accessible media sources to be a good resource for learning outside of the classroom when the source is credible, while 4.8% indicated no change in perspective. Many of these responses illustrate a shift in perspective as students were encouraged to actively critique media, recognize bias or framing that supports the spread of misinformation, and question credibility of sources through application of CML principles. Through critical reflection students described future intentions of utilizing more evidence-based evaluation strategies, reflecting competencies required for digital scientific communication (Brownell et al. 2013). A conceptual flow of themes observed in student reflections was developed to illustrate progression of media evaluation practices through the assignment (Fig. 1).

These findings suggest that engagement with CML principles in a structured assignment encourages students to reflect on their media evaluation practices in ways that align with key elements of transformative learning theory, emphasizing reflection on assumptions and perspectives (Mezirow 2000, 2018; Cranton 2023). Students’ awareness of bias, even unconscious bias, agenda-setting strategies, and the ease with which misinformation spreads suggests movement beyond superficial processes of evaluation as they engage in critical reflection on how information is produced, presented, and interpreted on public platforms. It is important to note that several students reported no change in how they will evaluate media, stating that they already approached it with a great deal of skepticism. This finding is still in line with transformative learning theory which states that at times, reinforcement of existing practices can occur (Cranton 2006). In this way, the project supported students at varying competency levels, strengthening critical evaluation skills among less experienced students, and validating the more evidence-based approach of experienced students.

Students emphasized the need for informed and credible science communicators in digital spaces, as they began to question veracity of widely accessible media content rather than accepting them at face value, or allowing emotion to influence their judgement. As one student stated,

I realized that I have become really reliant on learning things on social media without much thought that the information may not be reliable…I never thought much about the credibility of the sources I was using.

Students’ desire for more credible sources in easily accessible media highlights the broader challenge of science communication, where complex, dense, and nuanced research findings must compete with provocative or emotionally compelling storytelling with poorly supported claims (Brossard 2013). It is the responsibility of animal science-trained professionals to engage in public discourse. Effective scientific communication, as described by Brownell et al. (2013), requires not only accurate reporting of peer-reviewed, evidence-based research, but translating it into forms that are understandable, credible, and appealing to a diverse audience. In highlighting the need for informed creators, students are also recognizing how misinformation can flourish in the absence of credible voices, as evidenced in their descriptions of misleading, agenda-driven or unsupported claims found in easily accessible media. Transformative learning theory posits that such recognition may precipitate behavioral modification (Mezirow 2018), perhaps influencing how students view future professional roles including extension, outreach, or public communication roles, as viable options to apply scientific expertise beyond traditional laboratory or industry roles. The CML framework helps students to make connections between evaluating information, assessing credibility, and participating responsibly in digital communication environments. Together, these connections support the development of animal science graduates that are better equipped to navigate a complex and at times contentious digital environment.

Integrating a media literacy assignment into the Animal Nutrition curriculum helped students to identify agenda-setting and bias, recognize superficial credibility evaluation strategies, and engage in critical reflection aligned with transformative learning. Given the ubiquitousness of digital misinformation related to animal agriculture, embedding media literacy assignments into animal science curricula can support development of critical evaluation and communication competencies, thereby helping to prepare students for future careers in extension, veterinary medicine, or public-facing industry where the ability to evaluate and communicate credible information is increasingly relevant (Funk et al. 2016; Kellner and Share 2019; Woodside 2024).

## Conclusion

Through this project, students described increased attention to credibility, purpose, and bias when reflecting on how they engage with media. Engagement with the CML framework provided opportunities for reflection on how they engage with digital media, and how that information is produced, evaluated, and interpreted, consistent with key elements of transformative learning. These findings highlight the value of integrating structured media literacy activities into animal science curricula to support students in critically evaluating information. Given the prevalence of misinformation related to animal agriculture, developing these competencies in undergraduate students is particularly relevant for those entering public-facing roles where accurate interpretation and communication of information is essential.

Although this study focused on student evaluation of digital media sources, the rapid emergence of artificial intelligence (AI) tools and large language models introduce additional complexity to the study of media literacy. As data were largely collected prior to widespread adoption of generative AI tools, student reflections did not explicitly address AI-generated content. However, CML principles such as examining evidence, questioning assumptions and considering purpose and context of information remain applicable. Adapting these frameworks to address AI-generated content represents an important direction for future research and instructional design.

Embedding media literacy throughout animal science curricula can support development of students who are well prepared to navigate a rapidly evolving digital landscape and allow them to contribute responsibly and effectively to scientific discourse in the digital world.
